# Making the right connections: biological networks in the light of evolution

**DOI:** 10.1002/bies.200900043

**Published:** 2009-10

**Authors:** Christopher G Knight, John W Pinney

**Affiliations:** 1 Faculty of Life Sciences, The University of Manchester, Michael Smith BuildingOxford Road, Manchester M13 9PT, UK; 2 Centre for Bioinformatics, Division of Molecular Biosciences, Imperial College LondonLondon SW7 2AZ, UK

**Keywords:** correlation network, eQTL network, eurovision song contest, gene regulatory network, protein–protein interaction network

## Abstract

Our understanding of how evolution acts on biological networks remains patchy, as is our knowledge of how that action is best identified, modelled and understood. Starting with network structure and the evolution of protein–protein interaction networks, we briefly survey the ways in which network evolution is being addressed in the fields of systems biology, development and ecology. The approaches highlighted demonstrate a movement away from a focus on network topology towards a more integrated view, placing biological properties centre-stage. We argue that there remains great potential in a closer synergy between evolutionary biology and biological network analysis, although that may require the development of novel approaches and even different analogies for biological networks themselves.

## Introduction

**Figure 1:**
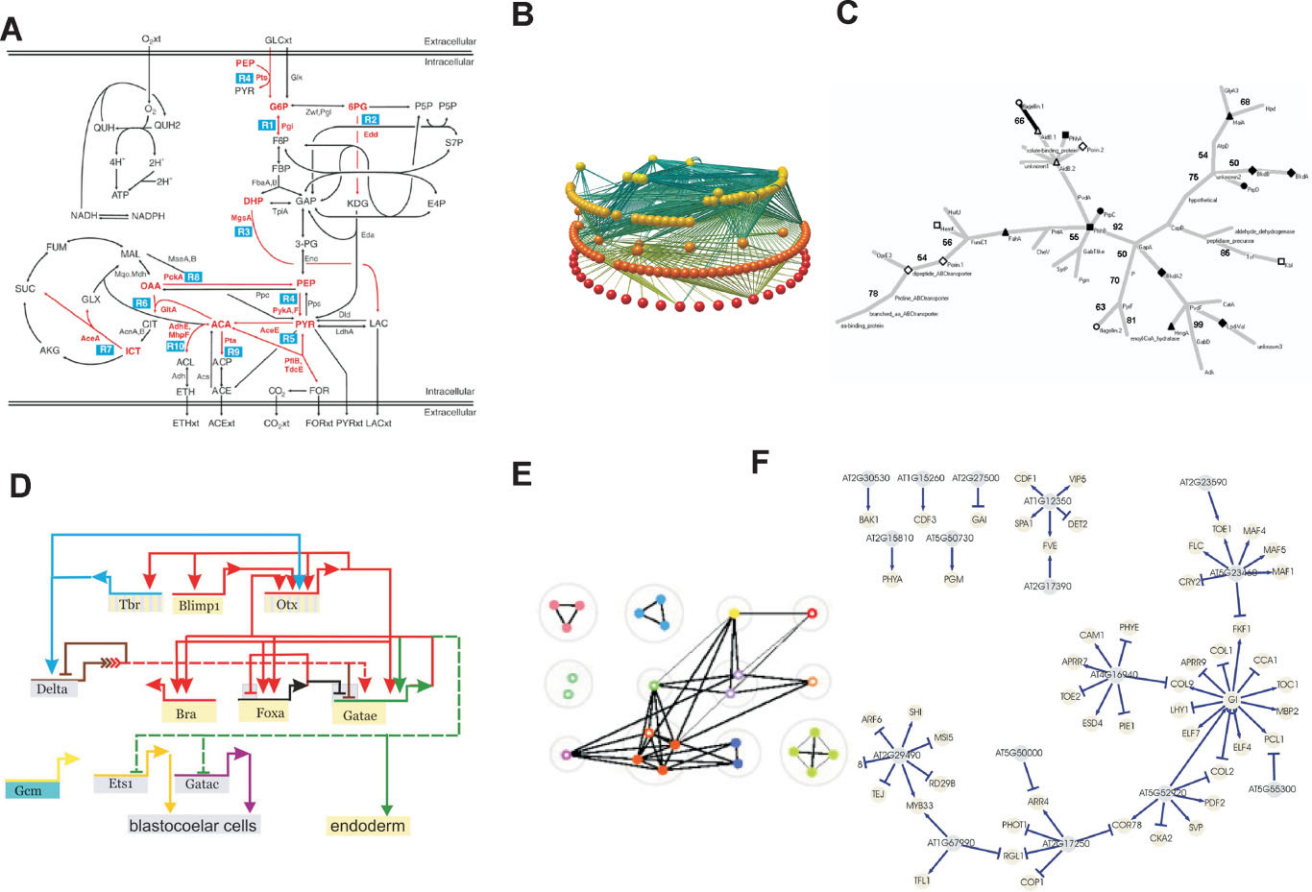
Recent examples of networks used in evolution-related studies in diverse areas. **A**: Metabolic network of central carbon metabolism in *E. coli*, as used for evaluating flux balance analysis (FBA) objective functions.^100^ **B**: Food-web network of species in the Burgess Shale.^3^ **C**: Correlation network of proteins affected in a bacterial experimental evolution.^72^ **D**: Gene regulatory network (GRN) for endomesodermal specification in sea star.^90^ **E**: Inferred ancestral chordate protein–protein interaction (PPI) network for bZIP transcription factors.^62^ **F**: Regulatory network of genes involved in the transition to flowering in *Arabidopsis* inferred from expression quantitative trait locus (eQTLs).^89^

Many diverse and distinct biological systems may be represented as networks (Fig. 1). It is perhaps reasonable to expect evolutionary processes to act on each in a different way. Yet common network representations encourage the use of common analytical tools and suggest the potential for cross-fertilisation of ideas and techniques. Studying evolution at the level of the network representations of biological systems may therefore provide a broad and unified view of evolution itself. Conversely, an evolutionary view may be just what is required to make sense of increasingly numerous, complex and confusing networks. Systems biology (SB), one of the main modern purveyors of network representations of biological systems, sets out to navigate between biological levels, molecular and functional, in a mathematically explicit way.^1^ It was to help navigate between such levels, molecular and organismic, that Dobzhansky coined the axiom ‘Nothing makes sense in biology except in the light of evolution’.^2^ With networks now being invoked in fields from paleobiology^3^ to human behaviour,^4^ the issue is shifting from the existence and identity of such networks to their biology and the insights they might provide into evolution at the systems level. It is therefore appropriate to ask how much evolutionary sense is being made of biological networks.

Others have considered the rise of network thinking in relation to evolution^5^ and the importance of network analyses in bridging the gap between evolutionary biology and other fields.^6^ Specific issues of modularity,^7^ robustness and evolvability^8^ and the related phenomena of pleiotropy and epistasis^9^ in relation to biological networks have been subjects of much interest. We necessarily encounter these themes, but focus mainly on existing paradigms of how networks evolve. We ask what can be understood about the biological mechanisms involved in network evolution and highlight emerging approaches that may help us find answers to this question.

## Network structure

The theory of complex networks and their properties has been extensively reviewed elsewhere, both generally^10^ and regarding their biological applications.^11^ Throughout the short history of this science, research has tended to focus on the description of a network's structure in terms of global summary statistics such as its observed degree (*i.e*. connectivity) distribution, mean clustering coefficient or characteristic path length. The most famous example is the power law (or ‘scale-free’) degree distribution, where the frequency of nodes having a degree *k* has the form 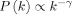, where *γ* > 1. Networks of this form have few nodes with many connections, but many nodes with only one or two connections. Power law behaviour in the structure of networks was first noted by Price in the patterns of citations in scientific publications^12^ and subsequently popularised by Barabasi and Albert, who observed power law distributions for network data concerning the World-Wide-Web, actor collaborations and a power grid.^13^ Subsequent work from this and other research groups reported power law distributions for many biological networks, both subcellular [*e.g*. metabolic^14^ and protein–protein interaction (PPI)^15^] and organismal.^4^ It is important to note, however, that the degree distribution alone is a poor encapsulation of a network's topology, since a given degree distribution may be satisfied by many different networks with substantially different architectures.^16^ Additionally, there are various alternative forms proposed for the degree distributions of biological networks. There is currently much controversy over which of these might in fact fit the observed network data the best,^17^ whether such distributions have any real biological significance,^18^ and to what extent the values of network summary statistics might be affected by noise,^19^ sampling^20^ or data handling.^21^

## Evolution of topology and beyond

**Figure 2:**
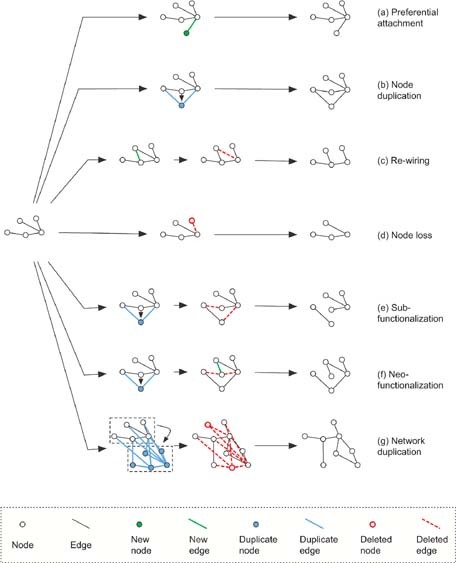
Illustration of some processes of network evolution. These processes range from **A**: the purely graph-theoretical concept of preferential attachment,^13^ via increasingly biologically motivated concepts of **B**: node duplication, **C**: re-wiring, **D**: node loss, **E**: sub-functionalization and **F**: neo-functionalization, to **G**: network duplication, analogous to a whole-genome duplication event.

When researchers started to address the question of how these networks evolved, belief in the primacy of the degree distribution led to a focus on evolutionary mechanisms that would generate power law networks.^22^ Just as there may be many plausible topological models to fit a particular degree distribution, there are many plausible stochastic models of network evolution that could generate a given topology.^23^ For example, the preferential attachment model (Fig. 2A)^13^ is one simple way to generate a power-law network by the progressive addition of nodes, where each new node is attached to an existing node with a probability related to the degree of that node. However, preferential attachment seems a particularly unreasonable mechanism for the evolution of many biological systems. Several biologically motivated schemes incorporating node duplication (Fig. 2B) and subsequent loss and/or gain of interactions (Fig. 2C) have been proposed.^24^,^25^

**Figure 3:**
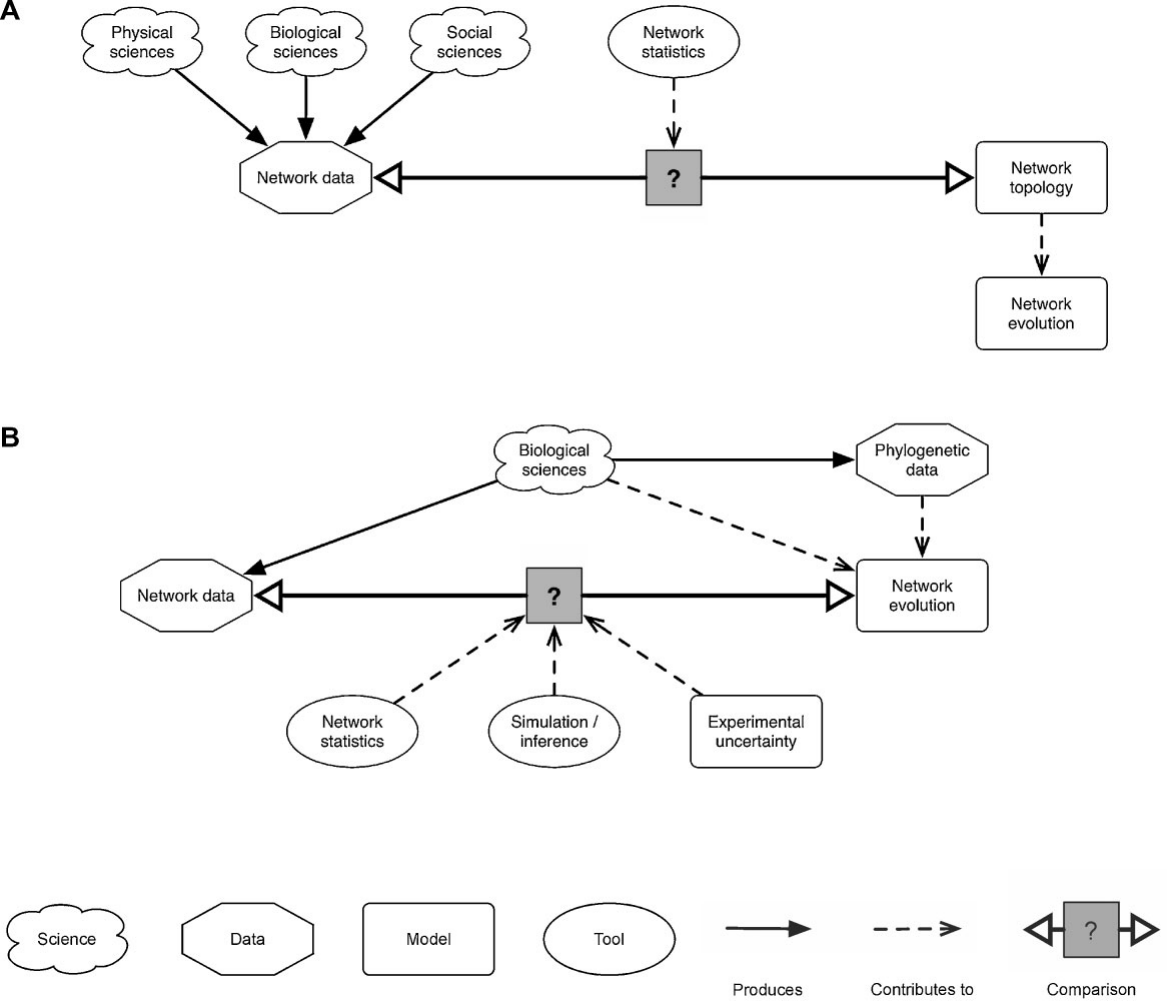
Changing research paradigms in the study of biological network evolution. **A**: Throughout the development of network theory, biological networks have been of great interest as data-sets to be analysed alongside examples of technological (*e.g*. internet, world-wide-web, power grid) and social (*e.g*. friendship, collaboration) networks. Early work tended to focus on the development of simple models of archetypal network topologies. Although many authors were keen to address the evolution of biological networks, the evolutionary models developed were primarily designed to reproduce the simple topologies under consideration, and as such were rarely tested directly against the data. **B**: A more sophisticated research paradigm for studying the evolution of biological networks starts from the viewpoint that any evolutionary model should relate directly to the biological system under study, with reference to population genetics and genomics where appropriate. Using simulation and probabilistic inference methods, models of network evolution can be tested directly against the biological data, taking factors such as experimental uncertainties and biases into account.

Much of this research into network evolution is typified by a paradigm in which a topological model is described, justified to some degree in its fit to observed network data, then subsequently discussed in terms of its evolutionary implications (Fig. 3A). However, in spite of its popularity, this approach appears to have produced few insights into the true evolutionary mechanisms that resulted in present-day biological networks. It also fails to incorporate existing understanding of evolutionary processes. For example, models of evolving PPI networks or gene regulatory networks (GRNs) may need to incorporate gene deletion (Fig. 2D),^26^ subfunctionalisation and/or neofunctionalisation of duplicated genes (Fig. 2E, F, )^27^ and whole-genome duplication (Fig. 2G).^28^ Models incorporating such processes can use computational simulation or inference methods to compare the model to the observed data, to fit parameter values and to compare alternative models in terms of their relative likelihoods.^29^ Choosing a biology-centric rather than topology-centric approach also allows incorporation of known information concerning evolution of the specific system, *e.g*. phylogenetic trees for the genes involved and the gene duplications and losses that may be inferred from them by cross-species analysis.^30^ As additional biological factors are implicated in the evolution of networks, it is possible to extend such models to include them. For example, the importance of population genetics in systems evolution has largely been ignored until now, but may prove to have a profound influence on network structures.^31^ A suggestion for how this alternative research paradigm might be structured is outlined in Fig. 3B. Instead of focussing on a particular theoretical network topology, this approach uses existing biological knowledge to build a realistic model for the evolution of the specific network being studied.

How then can such biology-centric approaches be applied to network evolution? We consider briefly three areas where answers to this question are being worked out: SB, evolution of development (‘evo-devo’) and ecology.

## Systems biology

As popularised by Kitano,^1^ SB concerns feedback of wet-lab and dry-lab experiments, putting together a mathematically explicit understanding of the structure and function of subcellular networks from their component parts. Since the challenge of constructing any such single network is great (*e.g*. those encapsulated by the KEGG databases^32^), evolutionary change has typically received only peripheral attention. Furthermore, SB has traditionally advocated applying engineering approaches to biological systems,^33^ which, while potentially useful for functional network analysis and synthetic biology,^34^ does not easily accommodate thinking about the evolutionary (as opposed to engineering design) processes that sculpt biological systems. Nonetheless, once constructed, SB network models are adaptable to different tasks, including providing insight into the processes of evolution which constructed them.^35^ Such an approach has proved useful in studying horizontal gene transfer,^36^ enzyme dispensability^37^ and minimised genomes.^38^

Evolutionary analysis of SB networks is a relatively new phenomenon; a longer-standing relationship of evolution to SB networks is as an experimental tool. An example is the use of evolution experiments in *Escherichia coli* to test metabolic network model predictions.^39^ This was successful in as much as bacteria evolved to the model-predicted phenotype. However, the actual evolutionary process whereby the cells evolve to the predicted optimum involves mutations to genes outside the network model.^40^ This implies that what natural selection identifies as ‘the system’ is not necessarily limited to specific networks (be they metabolic, transcriptional or at any other level). This finding – that evolution may define biological networks differently to biologists – is an important caveat to which we return below in the context of transcriptional networks.

Another insightful SB example in which evolution has been used as a tool involved experimentally ‘re-wiring’ a transcription network by re-pairing transcription factors and their promoters.^41^ This study shows the ability of transcriptional networks to tolerate substantial modification, but more importantly demonstrates an interaction (epistasis) between such introduced links and subsequent evolutionary changes: some added links seem consistently to enable more successful adaptation to stressful conditions than the wild-type network. The molecular basis of this evolution remains to be determined and could yield important insights into the relationship between transcriptional control networks and their targets. Such innovative SB approaches offer new ways to tackle old questions surrounding the nature of the evolutionary genetic phenomena of epistasis and pleiotropy.^42^

## Evo-devo

Transcription factor networks have also been a key area in evolutionary developmental biology, evo-devo.^43^ However, unlike in most SB studies, evolution of network structure has been a focus in understanding network models. Similarly, the nature of development imparts different emphases from SB in the network models developed, notably on spatial aspects.^44^ Evo-devo ideas have been developed in relation to biological network evolution, *e.g*. concerning homology,^45^ that may be relevant in other spheres of network evolution. For instance, in the paradigm of eye development there is homology across vast evolutionary distances at the levels of high-level function (photo-reception) and key genes (notably *Pax6*), yet very different patterns at the intermediate levels of morphology (insect *vs*. vertebrate eyes) and the GRN. To make sense of such patterns, the focus has to be on the historical continuity of networks through evolution^45^ and ‘developmental system drift’.^46^ The latter phenomenon refers to network structure changes with ultimate function remaining unaltered. It is identifiable in evolutionary network simulations^18^,^47^ and analyses^48^ and is reminiscent of ‘neutral network’ evolution seen, for instance, in *in silico* systems of nucleic acid evolution.^49^

The mechanics of such network evolution are, however, very difficult to test, given the large timescales of interspecific evolution typical of evo-devo, where we only have developmental details for a few tips in a large phylogenetic tree. Nonetheless, ‘micro evo-devo’ is progressing in this direction.^50^ Within-genus work in *Caenorhabditis* nematodes reveals quantitative changes within a signalling network, the overall phenotype remaining constant (changes are described as ‘cryptic’ since they are only apparent following experimental manipulation).^51^ At shorter evolutionary timescales again, cryptic quantitative evolution has been identified in *Caenorhabditis elegans* over the course of laboratory culture,^52^ and experimental evolution has been used.^53^ Thus, it may be possible to use the *C. elegans* model system to look at steps of developmental network evolution individually and experimentally, which so far has been done only for microbial networks (*e.g*.^40^). Are these concepts and approaches applicable outside development? Quite probably – in the simulations showing developmental system drift the phenotype used is one or more gene expression levels,^18^,^47^,^48^ which is as applicable to unicellular systems as development, and may be more tractable in simpler, experimental evolution systems. More generally, while apparent disconnections between evolutionary behaviour at genotypic, phenotypic and network levels may be particularly acute when the phenotype is as complex as an eye or vertebrate limb, there is no reason to believe that such relationships are any less subtle in simpler and potentially more experimentally tractable systems such as microbial metabolism. Indeed, similarly complex genetic relationships of orthology, paralogy and functional divergence undoubtedly exist, as evidenced in complete microbial genome sequences.^54^

## Ecology

Ecology has a long history of using networks – food webs (Fig. 1B) are some of the longest standing networks in any field (*e.g*. Briand collated 40 published webs over 25 years ago^55^). Like evo-devo, network studies in ecology have focused on evolution and the underlying biological (as opposed to purely graph-theoretical) processes. Similarly, network ‘dynamics’ refers to the evolution of structure over time (a sense in which it has also been used for PPI^28^ and transcriptional networks^56^ in contrast to the SB sense of the temporal kinetics of variables within a given network). However, the sorts of processes occurring among networks of biological taxa in ecological networks are scientifically rather distant from the subcellular networks considered so far, although arguably not as distant as the influential social, citation and world-wide-web network paradigms.^57^ For instance, an important distinguishing feature of ecological networks from either social or world-wide-web networks is variation in the abundance of the entities represented by network nodes.^57^ This feature has important implications for designing appropriate null models for network structure^58^ and is shared with, for instance, PPI networks. Taken to its limit, ecological network evolution is appropriately modelled *via* individual-based models.^59^ However, even when topology alone has been used, ways have been found to frame meaningful tests of underlying evolutionary processes.^60^

Ecology's subject matter means that evolutionary processes acting on the individual or population, notably natural selection, will typically act at or below the level of individual network nodes rather than at the level of the complete network or above, as with subcellular networks. In ecology this has led to clear distinctions being drawn between network structure evolution (especially *via* extinctions) and evolution of the components in the network (phylogeny), highlighting the relationship between the two.^61^ Such distinctions between levels of evolution are undoubtedly important in other systems, where, *e.g*. the distinction between the evolutionary history of a protein and the evolutionary history of its interactions may not be so obvious, but may be necessary to an understanding of what is going on in evolution (*e.g*.^62^). Ecological network evolution is also tackling the move from discrete (binary) networks to quantitative networks (weighted graphs showing the strength of interactions).^63^ In addition to representing biological aspects of the system, quantitative networks can be used to analyse the effect of sampling effort,^64^ something that is coming to prominence in PPI networks.^21^

## Discrete *versus* quantitative networks

The relationship of discrete ‘wiring diagram’ networks to quantitative (weighted) representations of the same biological systems is a current challenge for evolutionary network analysis across diverse fields. It is important firstly because the representation used affects the capture of evolutionarily important characteristics.^65^ Secondly, as demonstrated in evo-devo analyses of nematode vulva development highlighted above, what ends up as discrete network evolution over long (inter-generic) evolutionary timescales (*e.g*.^66^) may start as quantitative changes over shorter (intra-generic) timescales.^51^ Therefore, understanding the mechanisms of network change that occur in the individual steps of evolution will entail obtaining quantitative descriptions of the system, such as the proportion of different cell fate outcomes from signalling networks.^51^

SB has perhaps moved furthest down the road of exploring alternative discrete and quantitative models of the same or similar systems. Thus, constraint-based analyses of metabolic networks, notably flux balance analysis (FBA), may require little more than discrete wiring diagrams^67^ (Fig. 1A), but produce only limited steady-state predictions. In contrast, fully quantitative kinetic modelling provides a clearer view of quantities that are more easily measured in real systems (*e.g*. metabolite levels), but requires much more prior information,^68^ the acquisition of which is a substantial bottleneck (for instance the enzyme kinetic parameters stored in the Sabio-RK database^69^ are primarily culled from old literature and collected in highly varied conditions; high-throughput approaches using standardised conditions are only slowly being developed). This amounts to a trade-off between the requirement of discrete models for less detailed experimental knowledge and applicability to larger systems, and the greater, subtler and probably more realistic insight of quantitative models. This trade-off will become increasingly apparent across biological network analyses, where the network required to answer specific evolutionary questions will not necessarily be the most quantitative network available. Thus, so far in SB, primarily discrete FBA network analyses have been used effectively to tackle evolutionary questions (*e.g*.^38^).

## Phenomenology, null models and mechanism

We currently know so little about the phenomenology of network evolution that charting the ‘natural history’ of a network evolution will frequently be valuable in itself, without comparison to a null or expected change.^70^ The natural history task is non-trivial, firstly in terms of separating true evolutionary change from apparent changes due to technical error.^21^ Secondly, in our ignorance of ancestral network states, evolutionary reconstruction is a delicate exercise.^62^ Perhaps the neatest answer to the first problem is to concentrate on approaches to network evolution that compare networks with equivalent technical errors. Such a focus argues against non-comparative analyses, such as attempting to draw evolutionary inferences from topological analysis of any single PPI network (see above) and against comparisons of networks constructed from different studies carried out in different laboratories at different times. Conversely, this is an argument for focusing on networks identified and compared in a single study, as in nematode vulva specification studies,^51^ and the expression quantitative trait locus (eQTL) and correlation network examples discussed below. In a similar vein, the neatest solution to difficulties of phylogenetic reconstruction may be to focus on experimental systems where the ancestral states are observed directly, as in microbial experimental evolution systems^71^ (see below). Even when all these approaches are taken, the phenomenology of network evolution may remain challenging to unpick (*e.g*.^72^).

Focusing on single-study comparative and experimental approaches will help make valid evolutionary comparisons. However, the questions of the mechanisms and evolutionary forces underlying network evolution are clearly extremely important. Whether or not there is any empirical basis for doing so, evolutionary, and in particular adaptive, hypotheses of network change are the subject of widespread speculation.^73^ Full population genetic null models of network evolution would bring clarity to these issues, but are in their infancy and a bottleneck to progress in this direction.^31^ Nonetheless, simulations are yielding results, notably that non-adaptive evolution can result in pathway architectures more complex than strictly required for a selected function.^31^,^74^ Simulation also indicates the importance of considering the network level of evolution, rather than concentrating only on genotype and high-level phenotype.^6^ For instance, low within-population polymorphism at a single locus coupled with high between-population divergence might be interpreted as a signature of adaptive evolution. However, simulating the network context of a locus's evolution, that signature can be shown to arise in some cases simply from stabilising selection around a given phenotype.^18^ Such simulations are currently rather abstracted relative to experiment, but, with care, adaptive hypotheses may be testable in real networks.^75^

Crucial to unpicking the reality of the roles of adaptive and non-adaptive processes in network evolution will be analysis and comparison of experimentally evolved strains undergoing adaptive, or specifically non-adaptive, evolution. Mutation accumulation experiments consider non-adaptive evolution. The focus is typically on the nature and rates of mutation.^76^ Probing network effects experimentally (*e.g*. by genetic^77^ or physical^51^ manipulation) in mutation accumulation lines may therefore prove illuminating. For adaptive evolution, there is much more experimental evidence, not least from Lenski's paradigmatic long-term (now over 20 years) experimental evolution of *E. coli*.^78^ This has demonstrated that the networks (or network ‘modules’) where adaptive mutations lie can be very distinct from the selected phenotype. Thus, while selection principally concerns metabolic traits (growth on glucose as a sole carbon source), adaptive mutations are seen involving DNA superhelicity and the stringent response.^78^ The reverse situation is also seen in shorter-term evolution in another bacterium, with selection for a non-metabolic trait being effected by mutations in a gene principally controlling metabolism.^72^ In a social experimental evolution system,^79^ the wide diversity of genes where mutations can result in social cheating is of itself predicted to provide a route to evolving network complexity *via* ‘conflict-generated churning’.^80^ This raises important issues, beyond our scope here, around the role and meaning of pleiotropy and modularity in network evolution.^7^,^9^,^81^ In practice, unpredictability of the loci involved in adaptive evolution, even of relatively well understood networks, makes it difficult to use the ‘candidate gene’ approaches that have been successful in evo-devo for identifying the genetic basis of evolution (*e.g*.^82^). However, falling costs of complete genome sequencing are removing the need for a candidate gene when identifying small numbers of changes in experimentally evolved lines. This makes experimental adaptive evolution an increasingly promising area of research.

## Genetics, eQTLs and correlation networks

Beyond experimental evolution, the genetic basis of network evolution is harder to unpick. Evo-devo has a focus on transcriptional GRNs and within that, *cis*-acting regulatory changes.^83^ This focus has undoubtedly enabled insight into interspecific evolution of transcription-factor networks, particularly of key developmental systems.^83^ However, it is by no means clear that these are uniquely relevant genetic changes, even within developmental networks.^84^ Beyond development, transcription factors are in fact rather poorly represented among the genetic loci responsible for the evolution of yeast transcription.^85^

Whatever the scope of the evolutionary role of *cis*-acting regulatory changes, evo-devo expresses an important objective: to identify patterns among mutations with particular roles in evolution. At a broad level, some relationships have been identified, such as weak negative correlations between genes' evolutionary rate and the connectivity of their proteins in PPI networks.^86^ However, a much more interesting challenge will be to understand patterns of individual evolutionary steps at the molecular level. To obtain such an understanding, researchers will need to develop both global and probabilistic views of individual mutations in the context of biological networks. One approach to gaining such an understanding for expression is given by eQTLs.^87^

All quantitative trait locus (QTL) analyses link genotype and phenotype in evolution by assessing statistical associations between genetic loci that have evolved differences (markers) and some quantitative phenotype(s) of interest, typically as both segregate in a cross between evolutionarily diverged individuals. For eQTLs, the phenotypes of interest are transcription levels of genes. Since both genotype and phenotype in an eQTL association are defined by identifiable genetic loci, it is possible to construct a network where the nodes are genes and the directed and signed edges indicate that evolved changes at one locus are associated with transcription levels at the other locus^88^,^89^ (Fig. 1F), *i.e*. a network exclusively of evolutionary effects. Thus, while eQTL networks share with GRNs the superficial similarity of showing loci affecting one another's transcription, the approach is almost diametrically opposite – GRNs typically focus on evolutionary conservation (for instance the concept of evolutionarily conserved GRN kernels advanced by Hinman and Davidson^90^), whereas eQTL networks show only loci that change or whose expression is changed in evolution, typically over intraspecific evolutionary distances. Different approaches may be more appropriate for studying processes of network evolution at different evolutionary scales. The challenge will be to define how these views relate to one another and hence to obtain a global evolutionary view of relationships among molecules of the cell. Pursuing this end will complement and may provide insight into non-network studies that demonstrate the non-equivalence of evolutionary conservation and functional importance, with many genomic regions showing experimentally demonstrable functional roles without evolutionary constraint.^91^

QTL-based networks not only focus on evolution, they aim at a global unbiased view of relationships across a genome. Thus, they go some way towards identifying what biological networks are, as defined by evolution rather than biologists. This is an important issue in the light of the cases mentioned above, where the mutations underlying adaptive evolution occur outside the pre-defined network of the phenotype studied.^40^,^78^ In eQTL networks, the network *is* the evolution, limiting evolutionary comparisons among networks. An alternative approach without this issue, but similarly aiming at a global unbiased view, is correlation network analysis. Assessing the correlation of a wide class of cellular components, most commonly metabolites, across a series of perturbations, commonly the minor environmental changes seen across biological replicates, identifies some components as strongly correlated and others not. These correlations may be interpreted as a fully connected, weighted graph, typically represented as a simpler discrete network by a process of edge removal based on the strength or significance of the correlations^92^ (*e.g*. Fig. 1C).

Correlation networks represent co-regulation, rather than proximity in a biochemical network. For instance, metabolites adjacent in a metabolic network may not be the most closely correlated in a correlation network, an observation attested to experimentally^93^ as well as theoretically.^94^ Use of the term ‘network’ here is potentially problematic, in that its meaning is far looser than is usual for biological networks. While network graphs are generated to which graph theoretical approaches can be, and have been, applied, there is no direct physical interpretation of an edge in the graph in terms of a molecular interaction. However, correlation networks are very good for capturing and making sense of control relationships, potentially transient or otherwise elusive relationships, and how they change. For instance they have been successfully applied to understanding changes in currency markets.^95^ They have also been used to look at genetic changes^96^ including those changes responsible for adaptive evolution.^72^ However, studying the evolution of such graphs is tricky – it depends on how the fully connected, weighted graph is simplified, which may involve arbitrary thresholds, or approaches such as minimal spanning trees (MSTs, Fig. 1C). MSTs may be used as a clustering tool, equivalent to single linkage clustering.^95^ Thus the analysis of correlation networks merges into other, non-network analyses of correlation matrices. It may be that other such methods (*e.g*. approaches based on eigenvalues of correlation matrices^97^), while less visually striking and lacking the network buzzword, may be equally or more appropriate tools with which to tackle network evolution in terms of correlations among biological molecules.

Whether or not correlation networks are usefully classified as networks, they highlight an important point: in the evolution of biological networks, there are very general issues of approach that remain open. As we have seen, much current work stems from the popular concept of the cellular wiring diagram, and hence by implication the role of evolution is equivalent to that of re-wiring a radio.^33^ Many of the analytical techniques discussed above are firmly rooted in this metaphor, which may be applied to network evolution in more or less biologically reasonable ways (see Fig. 2). The wiring diagram as a biological analogy clearly has value, primarily in the clarity it brings, enabling the application of engineering approaches, both analytical and synthetic. However, its adequacy as a biological analogy is particularly questionable when it comes to evolution. Actual wiring diagrams are descriptions of how individual, static elements designed to perform specific functions – transistors, resistors, *etc*. – are arranged in a design to perform specific roles in a higher level function, *e.g*. transduction of radio waves into sound. It is a very static view with design and purpose inherent in the image. It may be possible to navigate such issues, but they are particularly problematic when it comes to understanding network evolution.^31^

The wiring-diagram analogy for biological systems was borrowed from one of many forms of network analysed in the physical sciences. A surprising subject for network analysis to emerge from the physical sciences more recently is the Eurovision song contest.^98^ We suggest that this may be a more appropriate analogy to borrow when it comes to the evolution of biological networks. The nature of the analogy is sketched in Table 1. Briefly, like an organism, the Eurovision song contest is a system that ‘works’, as evidenced by its survival. There is a role for the effectiveness of the individual network elements (countries, *cf*. biological molecules) at doing their apparent jobs in the system (producing music, *cf*. biological functions). But while necessary, these functions do not determine how the system as a whole functions or evolves over time. System function and evolution is underlain by a variety of relationships among the elements, of varying strengths and degrees of permanence. This implies that individual network changes (particular genetic changes in a cell, *cf*. switches among cliques of countries voting for each other in the Eurovision analogy) may be very difficult to attribute to specific causes and of limited significance in themselves. Nonetheless, analysis of network evolution remains a route to understanding system function (*e.g*. analysis of changes in Eurovision voting patterns successfully identified unofficial cliques of countries^98^).

**Table 1:** Comparison of analogies for subcellular biological networks

	Biological network	Wiring diagram	Eurovision song contest
**System**	Living organism	Electronic device (*e.g*. radio)	Music competition
**Elements**	Biological molecules	Electronic components	Countries' representatives
**Nature of elements**	Complex chemical entities with evolutionary histories	Minimal elements designed to perform simple tasks	Complex decision-making units with historical continuity
**Clearly defined element functions**	Performing biological functions (*e.g*. as described in GO terms)	Component specifications	Performing songs
**Other element features involved in network function**	Genetic location	None	Geographical proximity
	Transcriptional, translational, post-translational and degradation control		Politics
	More or less specific PPIs		Cultural history
	Other known and unknown relationships		Other unknown relationships
**Performance measure**	Inclusive fitness of an individual within a population	Performance of one or more pre-defined functions	International TV audience figures
**Evolutionary step**	DNA mutation	Addition or removal of connection or component	Change in individual voting behaviour

The analogy used for biological systems affects the sorts of questions, experiments and analyses expected to be most fruitful. Thus, the Eurovision analogy (Table 1), while less appropriate to engineering than a wiring diagram, presupposes a more dynamic and complex system. As with the Eurovision analysis itself,^98^ this suggests eschewing static approaches, such as degree distribution analysis, in favour of network analyses designed to deal with dynamically changing network topology. Thus, the Eurovision analogy fits better than the traditional one with dynamic evolutionary network phenomena discussed above such as developmental system drift^46^ and conflict-generated churning.^80^

## Conclusions

The application of network approaches to biological systems has gone through something of a hype cycle,^99^ where the ‘peak of inflated expectations’ corresponded to the widespread excitement, now passed, about scale-free networks and purely topology-based network approaches more generally, but a ‘plateau of productivity’ has not yet been reached. Part of the excess hype comprised the over-zealous application of ideas across very different fields, when the hypothesis (power-law degree distributions) was both weak and in many cases incorrect.^16^ However, that does not preclude useful cross-fertilisation of network ideas and techniques from different branches of biology. Research on the evolution of biological networks seems ideally placed to help steer a course between graph theory devoid of biological realism and experimental ‘ridiculogram’ networks devoid of theory.

In this essay we have highlighted some ways in which the evolution of biological networks is currently being studied, drawing on examples from diverse fields. It is clear that various issues of network evolution are common across these research areas, including challenges such as the need to move from discrete wiring diagram networks to quantitative weighted graphs, a change that may be assisted by moving away from the wiring diagram metaphor or analogy itself (Table 1). A major focus has to be on the genetic basis of network evolution and how to take as broad and unbiased a view as possible of the networks in question. The approaches highlighted show how an explicitly evolutionary view of biological networks helps us to draw links between fields and to frame wider questions about the meaning of biological networks. The paradigm of understanding biological networks caricatured in Fig. 3A, with evolution as a downstream appendix, is clearly inadequate. Alternatives are being developed. However, there is much work to be done before we reach the more tightly integrated paradigm envisioned in Fig. 3B, where we can make sense of biological networks in the light of their evolution.

## Abbreviations:

eQTL: expression quantitative trait locus
FBA: flux balance analysis
GRN: gene regulatory network
MST: minimal spanning tree
PPI: protein–protein interaction
QTL: quantitative trait locus
SB: systems biology
